# Impacts of Self-Efficacy on Food and Dietary Choices during the First COVID-19 Lockdown in China

**DOI:** 10.3390/foods11172668

**Published:** 2022-09-01

**Authors:** Wen Jiao, Matthew Tingchi Liu, Peter Johannes Schulz, Angela Chang

**Affiliations:** 1 Department of Communication, Faculty of Social Sciences, University of Macau, Macao 999078, China; 2 Department of Management and Marketing, Faculty of Business Administration, University of Macau, Macao 999078, China; 3 Institute of Communication and Health, Lugano University, 6900 Lugano, Switzerland; 4 Department of Communications and Media, School of Communication and Media, Ewha Womans University, Seoul 03760, Korea

**Keywords:** self-efficacy, dietary behavior, food consumption, socioeconomic status, mediating effects

## Abstract

The COVID-19 pandemic has caused a global public health emergency, increasing the prevalence of emotional distress, and potentially leading to altered diet behavior. Self-efficacy measures various aspects of perceiving and understanding emotions. The present study was carried out with the objective of understanding the effect of emotional self-efficacy on dietary behavior and quality. It also shed light on which elements contributed to the link between food-related behavior and perceived dietary quality during the first lockdown of the COVID-19 pandemic. Based on the factor analysis of nineteen food groups, choices, consumption, and socioeconomic status were examined in a sample of 441 Chinese participants. Multiple linear regression examined the association between food consumption, dietary quality, and self-efficacy. Contrary to prior research, the intake of salty snacks and alcoholic beverages dropped by 3.3% and 2.8%, respectively, during the first lockdown. Emotional self-efficacy negatively mediated the relationship between socioeconomic status and dietary quality. In conclusion, emotional self-efficacy is a well-established tool for evaluating how Chinese people cope with negative emotions. As an individual’s dietary quality was affected during the imposed lockdown, the present study offers valuable insight into psychosocial factors that may contribute to health disparities by advocating for organized nutritional support in future epidemic-related quarantines.

## 1. Introduction

The COVID-19 pandemic triggered a global public health emergency; this has increased the prevalence of emotional distress, potentially leading to altered diet behavior. When faced with restrictions such as working from home, lockdowns, and lack of social contact, people experience adverse affective outcomes [1,2,3]. The pandemic-induced quarantine during the COVID-19 outbreak was stressful, resulting in an increased daily intake of snacks and homemade meals [4]. Similarly, people exhibited changes in dietary behaviors with severe overconsumption of food occurring in response to negative emotional stimuli during the pandemic [5,6].

The world saw its first COVID-19 lockdown come into force in Wuhan, China [7]. China’s COVID-free policy tackled pandemic outbreaks in the following months with immediate lockdowns and swift mass testing. The social distancing and quarantines to prevent the spread of COVID-19 used in Wuhan became routinely employed in other major cities such as Beijing and Shanghai [8]. Many cities were effectively sealed off from the rest of the country.

Several studies carried out in Chinese populations have found negative changes in eating habits during the COVID-19 pandemic, including the increased intake of unhealthy snacks and high-calorie foods [9,10], the higher intake of preserved vegetables [11], and the decreased intake of seafood [12]. Despite previous studies attempting to explain the complex relationships between cognitive and emotional influence on eating behavior, limited evidence was generated on the mediating impacts concerning food choice and dietary behavior [13,14]. Our findings can inform dietitians and health professionals of these changes in time for better public health practice. In the following section, previous research on emotional self-efficacy and its scale development, the relationship between socioeconomic status and dietary quality, and the potential mediating impact of emotional self-efficacy are reviewed.

## 2. Literature Review

Self-efficacy refers to one’s belief in their ability to regulate negative emotional states when faced with adversity [15,16]. Emotional self-efficacy reflects one’s confidence in their ability to exert control over their motivation, behavior, and social environment toward health-oriented behavior [17,18,19]. The hierarchical process of emotional self-efficacy includes the perception, understanding, and expression of emotions [20] as well as the ability to control one’s emotional state [21]. When encountering risky situations, people with high emotional self-efficacy can cope with the adverse effects of affective sadness or fear reactions [22,23,24].

Emotional self-efficacy is marked by the ability to manage emotions internally rather than externally; very few studies have examined it as a screening tool on food choice and dietary quality. Instead, emotional self-efficacy was studied using the concepts of emotional intelligence and adaptive emotional functioning in the self and others in the existing literature (e.g., [16,20,21,24]). The trait of emotional intelligence focused on the quality of social interactions between the self and others was not applicable in our food and dietary quality study.

A critical social determinant of a sustainable healthy diet is socioeconomic status, a multifaced and all-encompassing construct reflecting an individual’s economic and social standing relative to others [25,26,27]. These studies have concluded that people with low socioeconomic status are more likely to choose inexpensive, high-calorie, and less nutrient-dense foods as their primary source of nutrition [28,29]. Conversely, people with high socioeconomic status are more connected to greater affluence and food access, leading to high dietary quality with nutrition adequacy [30,31,32].

Socioeconomic inequality is also reflected in the ability to cope with negative emotions [33]. Previous research has demonstrated that stressful low socioeconomic status environments reduce an individual’s reserve capacity to cope with psychological symptoms, making them more susceptible to negative emotions and self-perceptions [34,35,36]. Individuals with high socioeconomic status are more confident in their ability to control their negative emotions [37] and maintain emotional stability and intelligence [38]. Given the aforementioned concept of emotional self-efficacy [17,18,19,20,21], it is reasonable to expect a relationship between socioeconomic status and emotional self-efficacy.

Current dietary concerns during the COVID-19 pandemic include the overconsumption of calories but the underconsumption of whole grains, fruits, and vegetables by the Chinese (e.g., [39,40,41]). Since the early days of the COVID-19 pandemic, access to fresh food has been restricted, and people are spending more time at home. However, more time at home may have resulted in some positive habits including an increase in cooking. Considering that the effects of emotional self-efficacy on dietary quality are inadequately covered in the previous studies, the present study was carried out with the objective of understanding the effect of emotional self-efficacy on dietary behavior and diet quality. Food dietary patterns were considered and associations with other lifestyle factors were assessed. It also shed light on which elements contributed to the link between food-related behavior and perceived dietary quality during the first lockdown due to the COVID-19 pandemic.

Therefore, to fill these gaps, the present study aims to show the direction and presence of detailed relationships among an individual’s socioeconomic status, self-efficacy, and food intake and diet quality during the first COVID-19 lockdown. The following four hypotheses (H) were put forward:

**H1.**  *There were impacts of COVID-19 lockdown on food consumption patterns among Chinese adults in China.*

**H2.**  *Socioeconomic status could predict healthy dietary behavior, as well as emotional self-efficacy.*

**H3.**  *Emotional self-efficacy could predict dietary quality.*

**H4.**  *Emotional self-efficacy could be a mediator linking socioeconomic status and dietary quality.*

## 3. Materials and Methods

The Corona Cooking Survey (CCS), organized by researchers at the University of Antwerp (UAntwerp) in Belgium, is an international project for studying food, media, and society that began at the pandemic outbreak in 2020. A web-based questionnaire survey was designed to examine and compare the impact of the COVID-19 pandemic on food-related behavior before and during the COVID-19 pandemic [41,42]. A total of 67 question items were surveyed, including lockdown policies, shopping, cooking, and dietary behavior. The Ethics Committee from the Social Sciences and Humanities at UAntwerp approved the study (Approval No.: SHW_20_46). The questions from the CCS questionnaire required translation and back-translation into the local language. For consistency, updating of the items or adding questions was not allowed. A local survey was conducted from 17 April to 30 June 2020 using the university-sponsored software, Qualtrics XM platform (Qualtrics, Provo, UT, USA), for data collection.

### 3.1. Procedure

A pilot test for the Chinese participants was administered via the individual researcher’s network. The official survey was promoted via local popular online media, such as WeChat, QQ, and Sina Weibo, in order to reach diverse groups. Access to the questionnaire was available via mobile phones, tablets, or computers. The inclusion criteria comprised adults aged 18 or above who were native Chinese speakers and resided in Mainland China. In accordance with the CCS project, a standard consent form was prepared for the participants, granting researchers permission to conduct research on them. The agreement between the researcher and research participant outlined their respective roles and responsibilities throughout the entire research process. Before beginning the survey, participants in this study read the participant information sheet and had the opportunity to ask the researcher any questions. All participants were informed about the study, ticked the consent box, and provided their informed consent. Participation was voluntary and anonymous, and was met by spending at least 30 min completing the questionnaire; a bonus of USD 12.5 was offered to each participant as an incentive. A total of USD 250 was awarded to 20 participants from a draw.

### 3.2. Measurements

Nineteen questions assessed food type and consumption frequency. Concerning dietary patterns, previous studies provided a validated diet quality index to predict habitual food intake and nutrition information [42,43,44,45,46,47]. A high value for diet quality indicates positive dietary behavior across commonly recommended food groups. In this study, thirteen healthy foods and six unhealthy foods were adapted for the measurement.

Five items focused on socioeconomic characteristics, including education level, employment status, income loss, general financial struggles, and food purchase difficulties [48]. Variables such as gender, age, degree of closure measures, and self-reported lockdown time were considered as covariates in the analysis. All relevant measurements and scales are listed in Appendix A.

Nine questions measuring emotional self-efficacy in one’s ability to regulate negative emotional states were used [5,49,50]. Questions such as “I feel hopeless”, “I feel restless or fidgety”, and “I feel that everything requires effort” were listed. Feelings assessed were about worthlessness, nervousness, depression, and human connection to maximize the expression and emotion control. The factor analysis of emotional self-efficacy with factor loading and item—total correlation results are listed in Appendix B.

### 3.3. Data Processing

SPSS Statistics 24 and AMOS 21 (IBM Corporation, Armonk, NY, USA) were used. A descriptive analysis was conducted to examine the demographic characteristics of the samples. An explanatory factor analysis was conducted for reliability, while structural equation modeling (SEM) was used to evaluate the overall goodness of fit of the emotional self-efficacy model. Additionally, a K-means cluster analysis was used to classify groups of low and high self-efficacy, while the chi-square test of independence was performed to examine levels of emotional self-efficacy and the respondents’ characteristics.

A paired-samples *t*-test with a 95% confidential interval was adopted to compare the patterns of food consumption before and during the COVID-19 pandemic. Multiple linear regressions analyzed the mediator as an intermediary of the two variables; that is, socioeconomic status could affect dietary quality through the mediation of emotional self-efficacy.

## 4. Results

### 4.1. Descriptive Analysis

A total of 441 completed questionnaires were analyzed while 221 (32.4%) of incomplete questionnaires were treated as defective surveys. The sample included Chinese adults aged between 18 and 79 (M = 30.98, SD = 11.88), with most being female (62.4%, *n* = 275). The majority of the participants had a bachelor’s degree (38.8%, *n* = 171). The reported lockdown time was approximately 9.14 weeks. The unemployment rate was 32.4% (*n* = 143), while 57.8% of (*n* = 255) respondents reported income loss during the first lockdown in China. The level of financial struggles was high (M = 2.91, SD = 1.29), particularly with regard to difficulties in purchasing food (M = 2.78, SD = 1.41).

### 4.2. Emotional Self-Efficacy Scale

A Kaiser–Meyer–Olkin (KMO) test was conducted to measure sample adequacy. The merit of the factor analysis of KMO showed a value of 0.920, and the result of the Bartlett test of sphericity was significant (χ^2^ = 2195.17, *p* < 0.001). The cutoff criteria for KMO values indicated that values greater than 0.90 could be considered to have superb validity [51].

Validity was assessed using factor analysis, which yielded a single-factor solution (eigenvalue = 4.84, 69.10% of the variance explained). Seven items of the baseline for emotional self-efficacy in Chinese participants showed a high factor loading, ranging from 0.690 to 0.903. Previous studies indicated that factor loadings exceeding 0.70 were indicative of a well-defined structure (e.g., [52,53]).

The self-efficacy scale exhibited satisfactory internal consistency (Cronbach’s alpha = 0.925), which was higher than the acceptable reliability of 0.70. An acceptable item—total correlation ranged from 0.604 to 0.855, which came from seven questionnaire items. Moreover, the inter-item correlation matrix assessed the strength of the self-efficacy item as well as the direction of the relationship. The inter-item correlation matrix showed positive associations, ranging from 0.468 to 0.768 (*p* < 0.001). All results were higher than the minimum acceptance criteria of the rule of thumb (r = 0.30). The high and positive correlation values indicated that the items measured the same characteristics. Seven correlation values exceeding 0.70 illustrated a high extent of content homogeneity between two emotions: worthless feelings (item 3) and depression (item 5), hopeless (item 1) but restless (item 2), and nervous (item 4) while depressed (item 5). Table 1 shows the inter-item correlation of emotional self-efficacy among Chinese respondents.

**Figure 1:**
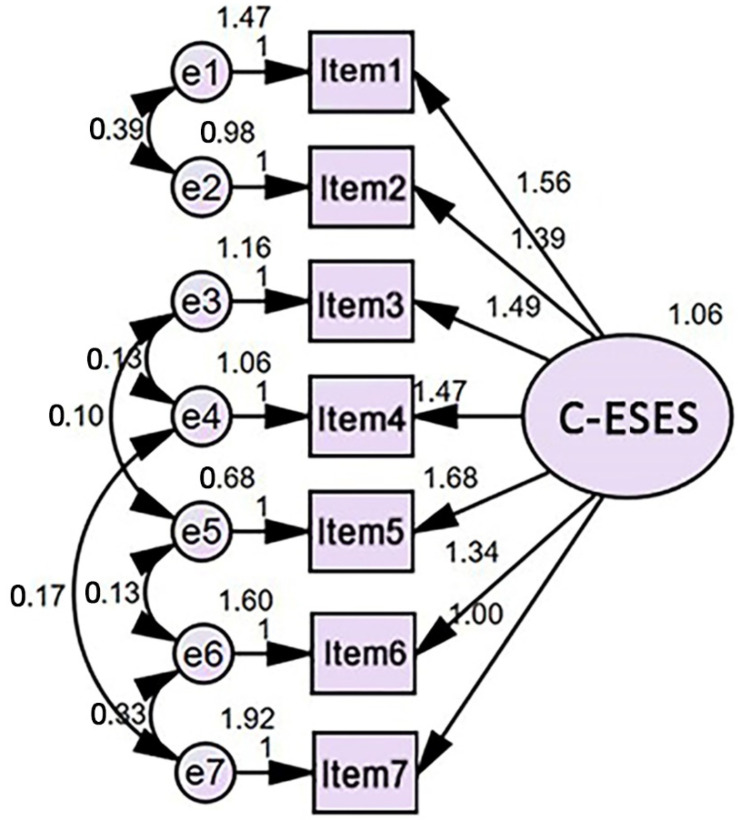
A statistical model for the emotional self-efficacy of Chinese respondents (C-ESES) based on the relationships among one latent variable (oval), seven measured items (rectangles), and seven corresponding unobservable errors (circles).

A structural model for parameter estimation was generated using AMOS. Under the 95% confidence interval, with the number of bootstrap samples being 5000 [54], four indices showed that emotional self-efficacy had a high baseline fit within a reasonable approximation error: goodness of fit index (GFI) = 0.992, comparative fit index (CFI) = 0.998, root mean square error of approximation (RMSEA) = 0.032, and Akaike information criterion (AIC) = 51.706. Figure 1 depicts the revised statistical model for the emotional self-efficacy of Chinese respondents (C-ESES). It was based on the relationships among one latent variable (oval), seven measured items (rectangles), and seven corresponding unobservable errors (circles).

The K-means cluster analysis showed that the proportion of participants in the low emotional self-efficacy group (52.2%, *n* = 230) was higher compared to that in the high emotional self-efficacy group (47.8%, *n* = 211). A chi-square testing independence was performed to determine the emotional self-efficacy levels of the participants’ demographic characteristics. This relationship was conditional but depending on whether the respondents had experienced income loss due to the COVID-19 pandemic (χ^2^ = 16.44, *p* < 0.001). In comparison, individuals with income loss had a lower emotional self-efficacy (67.0%, *n* = 154), while those without income loss had higher emotional self-efficacy (52.1%, *n* = 110). Table 2 shows a comparison of sociodemographic characteristics in two groups with various levels of emotional self-efficacy for Chinese respondents.

### 4.3. Food Choices

A paired-samples *t*-test was conducted to compare the type of food consumption during and prior to the first lockdown due to the COVID-19 pandemic. Consumption behavior with regard to food category did not change significantly during the first lockdown period. However, there were two exceptions: salty snacks and alcoholic beverages. The intake of salty snacks during the pandemic lockdown (M = 4.08, SD = 1.61) was lower compared to the time period of before the lockdown (M = 4.22, SD = 1.60), with statistical significance (*t*_440_ = −2.330, *p* = 0.020). A similar trend was found in the consumption of alcoholic beverages during the lockdown (M = 3.81, SD = 1.83) and prior to it (M = 3.92, SD = 1.84), with statistical significance (*t*_440_ = −1.968, *p* = 0.0497). Thus, H1 was partially supported. Table 3 lists changes in food consumption type during and prior to the first COVID-19 lockdown period among Chinese in China by using a paired *t*-test.

### 4.4. Socioeconomic Status and Self-Efficacy

A multiple regression analysis evaluated the outcome of self-efficacy, socioeconomic status, and food choices related to dietary quality. It revealed that socioeconomic status positively and directly predicted dietary quality (*β* = 0.094, *p* = 0.043). When socioeconomic status increased by 1 SD (SD = 1.04), an increase of 0.094 SD in dietary quality could be predicted. Thus, H2 regarding socioeconomic status could predict healthy dietary was supported.

In addition, socioeconomic status positively related to emotional self-efficacy (*β* = 0.143, *p* < 0.001). If socioeconomic status increased by 1 SD (SD = 1.04), an increase of 0.143 SD of emotional self-efficacy could be predicted. In other words, socioeconomic status had an impact on emotional self-efficacy. Thus, H2 regarding socioeconomic status could predict emotional self-efficacy was supported.

In line with this, the impact of emotional self-efficacy on dietary quality was found to be significant (*β* = −0.132, *p* = 0.022). Specifically, if emotional self-efficacy increased by 1 SD (SD = 1.55), a decrease of 0.132 SD of dietary quality could be predicted. Thus, H3 regarding whether emotional self-efficacy could predict dietary quality was supported.

Two significant results were observed between socioeconomic status and emotional self-efficacy (*β* = 0.143, *p* < 0.001), and emotional self-efficacy and dietary quality (*β* = −0.132, *p* = 0.022). In other words, emotional self-efficacy mediated the relationship between socioeconomic status and dietary quality (*β* = −0.019, *p* < 0.05). Specifically, if socioeconomic status increased by 1 SD (SD = 1.04), a decrease of 0.019 SD was found in dietary quality while it was mediated by emotional self-efficacy. Thus, the propositions of H4 regarding emotional self-efficacy being a potential mediator linking socioeconomic status and dietary quality was supported.

It is worth noting that the link between socioeconomic status and dietary quality (*β* = 0.094, *p* = 0.043) was stronger than when emotional self-efficacy was not considered (*β* = 0.075, *p* = 0.102) when considering the impact of emotional self-efficacy. Table 4 shows the multiple regression analysis for disparities in dietary quality, behavior, self-efficacy, and socioeconomic status.

## 5. Discussion

The study showed that 39.0% of Chinese individuals reported that their intake of healthy foods increased during the lockdown and 41.0% of them saw their intake of unhealthy foods decline. The unhealthy food consumption patterns of Chinese individuals during the initial lockdown was inconsistent with the previous studies (e.g., [9,10,11,12,55]). Despite people’s living standards improving and the pace of consumption upgrades accelerating in China, there were no significant changes in healthy food consumption during the COVID-19 pandemic.

Comparing the consumption of food category prior to the COVID-19 pandemic, fruits, vegetables, sugar-free beverages, and milk were still the main foods among Chinese. The salty snacks and alcoholic beverages decreased by 3.3% and 2.8%, respectively, and the remaining 17 food categories did not change dramatically. Alcoholic beverages were the least consumed item, followed by salty snacks and unprocessed fish.

From an outside perspective, could we really expect a lifelong set of tastes or habits to change when restrictions were imposed on people for weeks or months to contain a global health threat? It is very unlikely that this would happen, since this assessment is supported by our data analyses. The hypothesized effects and associations for explaining the changes induced by the pandemic proved to be non-existent, despite a few exceptions. This pertains to the hypothesis stating that the pandemic changes food consumption patterns and decreases unhealthy food groups.

Self-efficacy with emotional distress was measured to compare the perspectives of individuals with regard to the management of negative emotions during the COVID-19 epidemic. The analyses revealed significant relationships between the respondents with high socioeconomic status and those who were able to control their emotions (high emotional self-efficacy). An indirect effect of socioeconomic status might affect dietary quality, despite the fact that there was no significant effect reported. Moreover, when emotional self-efficacy was controlled, the direct effect of socioeconomic status on dietary quality was positive. In other words, self-efficacy played a mediating role in food choice and dietary behavior while considering socioeconomic status among Chinese individuals. This can guide future research aimed at elucidating the dynamic process of psychosocial constructs in behavioral outcomes.

This study’s findings intend to support practitioners in promoting the knowledge of dietary inequalities during the COVID-19 lockdown. Consistent with previous research (e.g., [28,29]), Chinese individuals with higher socioeconomic status engaged in healthier eating habits than those with lower socioeconomic status. Dietary disparities resulting from socioeconomic status could be mitigated by regulating negative emotions. In addition to emphasizing individual self-regulation, health practitioners should consider providing adequate psychological counseling and effective emotional support.

The research reported showed the effects of lockdown and the closing of many institutions of public life on food intake. There are quite a few other measures that might have affected people’s dieting during the pandemic. Irrespective of details on whether and when they were operative in China, the factors could include closing down bistros, unable to reach the food center in the workplace as the office was the home, more time for cooking due to lower working hours, shortages of food supply, less money available for buying food, etc. [56]. There are reasons why eating habits should change in a lockdown situation. When considering factors affecting dietary habits in general, dieting can be seen as a consequence of our education, family habits, a spouse’s preferences and tastes, and the persuasiveness of food marketing, etc. [57].

Several limitations should be noted. First, the present study survey was an international collaborative research project [41,42], and many study limitations existed due to constraints on research design, methodology, and materials. For instance, dietary intake was assessed by self-report without considering racial and cultural disparities.

Secondly, the web-based sampling method may limit the generalizability of the results. To mitigate the problem, the sampling method attempted to cover the adult group in various provinces during the first lockdown. Future studies are suggested to go through a stratified sampling method with specific age groups in cities with imposed lockdowns.

Thirdly, the effect of questionnaire length on response quality should be noted. The CCS questionnaire was lengthy, so the average time it took for a respondent to complete the entire questionnaire was at least 35 min. Thus, a revised version with concise but shorter questions for cross-cultural comparison should be taken into consideration.

Lastly, we argue for that the role of emotional self-efficacy that affects dietary behavior and quality along with food literacy in this relationship [23,58]. The potential impact of food literacy is to emphasize the variety of skills and knowledge required to choose and prepare food as well as to make appropriate decisions about a healthy diet [59,60]. Therefore, this study acknowledges the important role of food literacy by considering the contingency of emotional self-efficacy at subsequent stages of evolution.

## 6. Conclusions

As adherence to dietary patterns has been shown to be associated with health outcomes, the present study offers valuable insight into psychological and environment factors that contribute to health disparities in the Chinese. The emotional self-efficacy is a valid and reliable tool for measuring self-perceived ability of individuals to regulate negative emotions during the lockdown. Chinese individuals’ emotional self-efficacy was significantly affected by income loss, compared with other socioeconomic characteristics. In the context of the COVID-19 pandemic, Chinese eating habits have undergone minor changes, especially for salty snacks and alcoholic beverages.

The result also presented a causal process model by linking socioeconomic status with dietary quality through emotional self-efficacy. It explained the prediction power of emotional self-efficacy on dietary quality and food choice. The mediated relationship between food consumption, dietary quality, and emotional self-efficacy was supported. Another way to think about a mediator variable is that it carries an effect: emotional self-efficacy negatively mediated the relationship between socioeconomic status and dietary quality.

In conclusion, emotional self-efficacy was a well-established tool for evaluating how Chinese people cope with negative emotions. This study can enhance the researchers as well as health practitioners in understanding the complex mechanisms of self-efficacy, food choices, and dietary quality.

## Figures and Tables

**Table 1:** Inter-item correlation of emotional self-efficacy among Chinese respondents.

Item	1	2	3	4	5	6	7
1. I feel hopeless	1						
2. I feel restless or fidgety	0.768	1					
3. I feel worthless	0.648	0.665	1				
4. I feel nervous	0.659	0.687	0.715	1			
5. I feel so depressed	0.712	0.738	0.771	0.752	1		
6. I feel I struggle financially	0.606	0.609	0.624	0.581	0.704	1	
7. I feel more connected than usual	0.523	0.507	0.468	0.539	0.532	0.538	1

Note: Significance at the *p* < 0.001 probability level for all cells (two-tailed test).

**Table 2:** A comparison of sociodemographic characteristics with low and high emotional self-efficacy for Chinese respondents.

	Emotional Self-Efficacy		
Variable	Low	High	χ^2^
	230 (%)	211 (%)	
**Gender**			
Female	139 (60.4)	136 (64.5)	0.76
Male	91 (39.6)	75 (35.5)	
**Highest education**			
Below high school diploma	18 (7.8)	25 (11.8)	9.32
High school diploma or equivalent	92 (40.0)	57 (27.0)	
Bachelor’s degree	80 (34.8)	91 (43.1)	
Master’s degree	36 (15.7)	34 (16.1)	
Doctorate	4 (1.7)	4 (1.9)	
**Employment status**			
Work	163 (70.9)	135 (64.0)	2.38
No work	67 (29.1)	76 (36.0)	
**Income loss due to COVID-19**			
Yes	154 (67.0)	101 (47.9)	16.44 ***
No	76 (33.0)	110 (52.1)	

Note: *** *p* < 0.001.

**Table 3:** A comparison of change in food consumption type during and prior to the first COVID-19 lockdown period among Chinese in China by using a paired *t*-test.

Category	M (SD)		*t*_440_
	During	Before	
**Healthy food**			
Fruit	5.03 (1.56)	5.02 (1.52)	0.317
Vegetables	5.03 (1.39)	5.08 (1.42)	−0.966
Legumes/pulses	4.56 (1.40)	4.59 (1.34)	−0.585
Unsalted nuts or nut spread	4.41 (1.65)	4.43 (1.54)	−0.385
Unprocessed fish	4.17 (1.56)	4.15 (1.60)	0.435
Unprocessed poultry	4.20 (1.59)	4.15 (1.62)	0.974
Unprocessed red meat	4.28 (1.63)	4.35 (1.63)	−1.356
Unprocessed vegetarian alternative	4.42 (1.62)	4.42 (1.60)	0.118
Whole wheat	4.29 (1.61)	4.38 (1.56)	−1.576
Milk	4.67 (1.47)	4.62 (1.42)	0.913
Other dairy products	4.56 (1.52)	4.56 (1.53)	0.041
Plant-based drinks	4.26 (1.63)	4.29 (1.65)	−0.596
Non-sugared beverages	4.90 (1.66)	4.87 (1.65)	0.504
**Unhealthy food**			
Processed meat	4.35 (1.66)	4.41 (1.68)	−1.048
Sweet snacks	4.26 (1.60)	4.33 (1.51)	−1.281
Salty snacks	4.08 (1.61)	4.22 (1.60)	−2.330 *
White wheat	4.36 (1.72)	4.37 (1.65)	−0.079
Sugared beverages	4.25 (1.66)	4.18 (1.60)	1.221
Alcoholic beverages	3.81 (1.83)	3.92 (1.84)	−1.968 *

Note: * *p* < 0.05.

**Table 4:** Multiple regression analysis for disparities in dietary quality, behavior, self-efficacy, and socioeconomic status.

	Standardized Effect (β)		
Variable	Dietary Quality	Emotional Self-Efficacy	Dietary Quality (Total Effect)
**Control block**			
Gender	−0.080	0.000	−0.080
Age ^a^	0.111 *	0.213 ***	0.083
Degree of closure measures	0.108 *	0.187 ***	0.084
Self-reported lockdown time ^a^	0.000	0.176 ***	−0.023
Food choices influenced by marketing	0.336 ***	−0.280 ***	0.373 ***
**Prediction block**			
Socioeconomic status	0.094 *	0.143 ***	0.075
Emotional self-efficacy	−0.132 *	_	_
**Explanatory power**			
*R*-squared	0.137	0.399	0.126
*F*-value	9.791 ***	47.992 ***	10.442 ***

Note: * *p* < 0.05, *** *p* < 0.001; ^a^ means transformation by lg when entering regressions.

## Data Availability

The data are not publicly available due to the CCS project agreement and funding requirements. Derived data supporting the findings of this study are available from the corresponding author (Angela Chang) upon reasonable request.

## Appendix A

Detailed measurements and scales of variables.

**Emotional Self-Efficacy** “How do you feel during the COVID-19 pandemic?” (Likert scale: 1 = Never; 7 = All the time) - I feel hopeless - I feel restless or fidgety - I feel that everything requires effort - I feel worthless - I feel nervous - I feel so depressed that nothing could cheer me up - I feel I have more time than usual - I feel I struggle financially - I feel more connected than usual A higher average reverse scoring indicated a higher emotional self-efficacy degree. **Diet Quality Index before/during the COVID-19 Lockdown** “How often did/do you eat the following (portions of) foods?” (1 = Almost never; 7 = 2x or more times a day) **Healthy food** - Fruit (fresh or frozen) - Vegetables (fresh or frozen) - Legumes/pulses (e.g., beans, lentils, chickpeas) - Nuts or nut spread (unsalted) - Unprocessed fish - Unprocessed poultry - Unprocessed red meat - Unprocessed vegetarian alternatives (e.g., tofu, tempeh, seitan) - Whole wheat - Milk - Other dairy products (e.g., yoghurt, cheese) - Plant-based drinks (e.g., almond, oat, soy, rice) - Non-sugared beverages (e.g., water, coffee, tea) A higher average score indicated a higher degree of healthy eating. **Unhealthy food** - Processed meat/poultry/fish/vegetarian alternatives - Sweet snacks (e.g., sweets, cookies, cakes, pies) - Salty snacks (e.g., crisps, salted nuts) - White wheat - Sugared beverages (e.g., soft drinks, sugared coffee/tea) - Alcoholic beverages A higher average score indicated a higher degree of unhealthy eating. **Socioeconomic Status** **Highest education** - Below high school diploma (1) - High school diploma or equivalent (2) - Bachelor’s degree (3) - Master’s degree (4) - Doctorate (5) **Employment status** - No work (0) - Work (1) **Income loss** “Have you lost (a part of your) income since the lockdown?” - Yes (0) - No (1) **Financial struggles for general situation** “In general, how often is it a struggle to make your money last until the end of the month/payday?” - Never (1) - Very rarely (2) - Rarely (3) - Sometimes (4) - Frequently (5) - Very frequently (6) - Every time (7) **Food purchase difficulties** “In general, how often is it a struggle to have enough money to go shopping for food?” - Never (1) - Very rarely (2) - Rarely (3) - Sometimes (4) - Frequently (5) - Very frequently (6) - Every time I go shopping for food (7) **Control Variables** **Gender** - Female (0) - Male (1) **Age** (Ranging from 18 to 120) **Degree of closure measures** “Which of the following lockdown measures are currently in place?” (Multiple choice: 0 = No; 1 = Yes) - Events are suspended - Restaurants are closed for dining in - Bars and pubs are closed - Most non-essential shops are closed - Schools are closed - Public gatherings are prohibited (not allowed) - Public gatherings are restricted (allowed under restrictions) - If possible, people need to work from home - People in elderly homes are not allowed visitors/ only a restricted number of visitors - Non-essential movement is banned - Private gatherings are prohibited (people cannot visit other people) - Private gatherings are restricted (people can visit other people, but under restrictions) - Country borders are closed - Non-essential production has stopped - Face masks are mandatory in public **Self-reported lockdown time** “How many weeks have you been in lockdown?” (Ranging from 1 to 50) **Food choices influenced by marketing** “At the moment (during the lockdown), how often food advertisements or marketing influence your food choices when you go grocery shopping?” - Never (1) - Very Rarely (2) - Rarely (3) - Sometimes (4) - Frequently (5) - Very frequently (6) - Every time I go grocery shopping (7)

## Appendix B

**Table A1:** Earlier factor analysis of C-ESES with factor loading and item—total correlation results.

Item	Factor Loadings		Communalities	Item-Total Correlation	α, If Item Deleted	Screening Items
	Component 1	Component 2				
1. I feel hopeless	*0.823*	−0.254	0.741	0.663	0.896	Retain
2. I feel restless or fidgety	*0.850*	−0.165	0.749	0.690	0.893	Retain
3. I feel that everything requires effort	0.501	*0.651*	0.675	0.309	0.916	Exclude
4. I feel worthless	*0.818*	−0.300	0.758	0.656	0.896	Retain
5. I feel nervous	*0.847*	−0.112	0.730	0.662	0.893	Retain
6. I feel so depressed	*0.880*	−0.237	0.831	0.755	0.890	Retain
7. I feel I have more time than usual	0.574	*0.632*	0.729	0.399	0.912	Exclude
8. I feel I struggle financially	*0.803*	−0.011	0.644	0.573	0.896	Retain
9. I feel more connected than usual	*0.722*	0.301	0.611	0.476	0.902	Retain

Note: Eigenvalue 1 = 5.31; Eigenvalue 2 = 1.16; Cumulative variance explained 71.89%; Italic values indicate component attribution.

**Table A2:** Updated factor analysis of C-ESES with factor loading and item—total correlation results.

Item	Mean	SD	Factor Loadings	Communalities	Item-Total Correlation	α, If Item Deleted
1. I feel hopeless	4.68	2.01	0.849	0.722	0.785	0.911
2. I feel restless or fidgety	4.51	1.74	0.861	0.741	0.802	0.910
3. I feel worthless	4.83	1.88	0.847	0.717	0.781	0.911
4. I feel nervous	4.21	1.83	0.853	0.727	0.790	0.910
5. I feel so depressed	4.56	1.92	0.903	0.815	0.855	0.903
6. I feel I struggle financially	4.39	1.87	0.800	0.640	0.727	0.917
7. I feel more connected than usual	4.01	1.72	0.690	0.476	0.604	0.928

Note: Eigenvalue 1 = 4.84; Cumulative variance explained 69.10%.
